# Why Do People With Schizophrenia Exercise? A Mixed Methods Analysis Among Community Dwelling Regular Exercisers

**DOI:** 10.3389/fpsyt.2018.00596

**Published:** 2018-11-13

**Authors:** Patrick A. Ho, Danielle N. Dahle, Douglas L. Noordsy

**Affiliations:** ^1^Department of Psychiatry, Geisel School of Medicine Dartmouth, Hanover, NH, United States; ^2^Harvard Medical School, Division of Psychotic Disorders, McLean Hospital, Belmont, MA, United States; ^3^Department of Psychiatry and Behavioral Sciences, Stanford University School of Medicine, Stanford University, Stanford, CA, United States

**Keywords:** schizophrenia, exercise, lifestyle psychiatry, psychiatry, community dwelling adults with schizophrenia, qualitative analysis

## Abstract

Individuals with schizophrenia have reduced rates of physical activity, yet substantial proportions do engage in independent and regular exercise. Previous studies have shown improvement in symptoms and cognitive function in response to supervised exercise programs in people with schizophrenia. There is little data on motivations of individuals who exercise independently, or their chosen type, duration, or setting of exercise. This study explores motivational parameters and subjective experiences associated with sustained, independent exercise in outpatients with a diagnosis of schizophrenia or schizoaffective disorder. Participants completed a semi-structured interview and then were given a prospective survey containing visual analog scales of symptom severity and the Subjective Exercise Experiences Scales to complete immediately before and after three sessions of exercise. Results from the semi-structured interview were analyzed by modified content analysis. The most important reason for exercise was self-image, followed closely by psychological and physical health. Among psychological effects, participants reported exercise was most helpful for mood and cognitive symptoms. The prospective ratings demonstrated 10–15% average improvements in global well-being, energy, and negative, cognitive and mood symptoms, with almost no change in psychosis, after individual exercise sessions. This suggests that non-psychotic parameters are more susceptible to inter-session decay of exercise effects, which may reinforce continued exercise participation.

## Introduction

The benefits of exercise are well-established and include not only weight loss and improved cardiovascular fitness, but also a reduction in risk of early mortality and cognitive decline (1, 2). Exercise also has the potential to prevent or delay the onset of several mental health disorders (3, 4). More recent studies have shown that exercise can have therapeutic effects for patients with psychiatric disorders and can specifically help to control and reduce the symptoms of schizophrenia (4, 5). Randomized controlled trials (RCTs) have identified that exercise significantly improves negative symptoms (such as social withdrawal, anergia, or apathy) but few studies have shown an improvement in positive symptoms (most notably delusions or hallucinations) of schizophrenia (6–8). A recent review of 10 RCTs found significant improvement in global cognition among those receiving an exercise intervention, with medium to large effect sizes for improvements in working memory, social cognition and attention (9). Recent designs have turned toward combining exercise with cognitive rehabilitation in an attempt to magnify effects (10). Some studies have identified release of neurotrophic factors, changes in regional or global brain volume and even preservation of telomere length and integrity as possible biological underpinnings of these benefits (11).

As noted by Farholm and Sorenson, physical activity may be quite challenging for those with severe mental illness due to several barriers such as medication side effects, symptoms of mental illness, lack of support and even motivation (12). While many studies have addressed the other barriers, relatively few studies address motivation. A recent systematic review and meta-analysis of patients with any severe mental illnesses (such as schizophrenia, schizoaffective disorder, bipolar disorder, major depressive disorder, etc.) found that 91% of the participants were motivated to exercise to “improve health” with the most common response being “losing weight.” “Improving mood” and “reducing stress” were other common responses. These same patients also identified many barriers to participating in physical activity (13). When the motivations for exercise of people suffering specifically from “early psychosis” were examined, improving health also stood out as the main motivator for exercise. Interestingly, this subpopulation did not identify weight loss as the top motivator for exercise, as “increasing fitness/energy” was the top response followed by “taking your mind off things” and “being more confident in a gym.” The author points out that weight loss may not be a realistic goal in the short term, and it is encouraging that there are other more important motivators for physical activity (14).

Existing study designs have typically evaluated the addition of a time-limited exercise regimen to the routines of patients with schizophrenia who live a sedentary lifestyle. A recent meta-analysis by Vancampfort found that half of patients with severe mental illness do not get more than 150 min of moderate aerobic exercise per week (15). Conversely, this analysis indicates that there is a substantial subset of patients with severe mental illness who do exercise regularly. To our knowledge, no research has been conducted on the subset of patients with schizophrenia who have independently and spontaneously incorporated exercise into their lifestyles.

There are several advantages of exercise over other modes of therapy. First, exercise can lead to improvement in both the individual's physical and mental health. Second, exercise is the only available intervention in schizophrenia, a disorder associated with atrophic brain changes, which has clear and sustained neurotrophic effects (16). Additionally it is available to individuals who are apprehensive about taking medications or have contraindications to medications. It requires no equipment or access to a provider. It can be done at any time convenient to the individual and can be varied to meet the individual's specific needs or tastes. It requires only time, and can provide structured activity to a daily schedule. Finally it can be used as an adjunctive therapy that may allow the individual to minimize the use of other modes of therapy such as medications or to combat side effects associated with anti-psychotic medications such as weight gain (17).

There is currently very little evidence to guide clinicians in methods to support individuals with schizophrenia engaging in regular exercise (18). Furthermore, most studies lack data on the subjective experience of participants or how an individual's motivation to exercise can be sustained beyond the end of the study intervention. This study used a semi-structured interview to explore factors motivating sustained, independent exercise in a population of individuals with a diagnosis of schizophrenia spectrum disorder and prospectively evaluated the mental health effects of individual sessions of exercise in these individuals.

## Methods

The project received approval from the university institutional review board. The investigators identified eligible participants from throughout the clinical care sites of a medical center in rural New Hampshire and Vermont. Participants were drawn from the authors' usual care clinics for people with schizophrenia spectrum disorders including a residential treatment program and a community mental health center. Encouragement of a healthy lifestyle was routinely incorporated into care, but treatment was otherwise typical for patients with schizophrenia. Inclusion criteria for participating were being of age 18 years or older, with a diagnosis of schizophrenia, schizoaffective, or schizophreniform disorder, and exercising spontaneously for at least 30 min, three times a week, for at least a month. Participants could have co-morbid diagnoses, such as depression or substance use disorder. Participants were taking a range of commonly prescribed antipsychotic medications including risperidone, clozapine, olanzapine, and aripiprazole. Few were taking first generation agents. Exclusion criteria were irregular exercise or physical activity that did not meet definition of exercise (e.g., hyperactivity that is not planned or purposeful or whose objective is not improving physical fitness *per se*) and inability to read or write in English.

Informed consent was obtained from all potential participants prior to screening with the modified SCID. Once an eligible diagnosis was confirmed, participants completed a semi-structured interview following a template with one of the investigators (DND or DLN). The interview included demographic information, as well as, open-ended questions regarding the reasons for exercise, whether exercise helped manage symptoms, what participants do to motivate themselves to exercise, and information on exercise type, frequency, duration, and interruptions. The investigators then reviewed the instructions for participant self-rating before and after three future sessions of exercise. After reviewing how to complete the scales, participants were given 3 sets of pre- and post-exercise scales with a stamped envelope and offered a $10 gift card to return the study materials to investigators once completed. Laboratory and medication data were collected by chart review using the most recent available results.

In order to assess each patient's experience with exercise, we selected the Subjective Exercise Experience Scale (19) (SEES), a 12-item self-report scale assessing three general categories of subjective responses to exercise stimuli: positive well-being (e.g., great), psychological distress (e.g., miserable), and fatigue (e.g., tired). For each item on the SEES, participants rate how strongly they are experiencing each feeling state along a 7-point Likert scale, ranging from 1 (not at all) to 7 (very much so). The SEES has been shown to have high internal consistency across a variety of populations.

Given the lack of an existing psychosis self-report scale, we also created a simple scale using a visual analog format to capture each participant's experience of well-being and symptoms in their natural exercise environment, which we named the Noordsy-Dahle Subjective Experience Scale (NDSE). The NDSE asked participants to rate the present status of their symptoms and wellbeing on a 10 cm line. The NDSE was constructed with 2 items each for psychosis (hallucinations, delusions), negative symptoms (motivation, social interest), mood (depression, anxiety) and cognition (clarity of thought, concentration). Global well-being was measured with the well-validated Lehman Quality of Life (QOL) scale (20), and an additional item rating energy, a dimension identified by many patients as central to their well-being (21). A detailed description of each item was provided, as well as, anchors at each end of the visual analog line. For psychosis and mood items, 0 corresponded to “none,” and 10 to “extreme.” For motivation, social interest, cognition and energy items, 0 was low and 10 was high. On the global QOL item, 0 corresponded to “delighted,” and 10 to “terrible.” Mean pre-workout scores on the NDSE were calculated for each item to provide a reference baseline.

The data collected from the semi-structured interview was analyzed by modified content analysis. Participant answers were categorized using a priori categories included in the interview template (available on request) but were also searched for any emerging themes that were not hypothesized prior to the interview. Responses were coded and collated by theme and frequencies calculated. The data collected on the NDSE was converted into numerical values by measuring the point marked by the participant from 0 to 10 cm along the visual analog line. These values and the numerical values from the SEES were used to calculate the change from pre- to post-exercise for each item in each episode of exercise rated and then the mean change in each item across all exercise sessions was calculated.

## Results

Twenty-three participants were enrolled in the study and completed the semi-structured interview. Participants had a mean age of 37.6 years (standard deviation 13.5 years, range 18–67 years). Seventy percent were male, 30% were female and there was an average duration of illness of 15.4 years. Fourteen participants (61%) returned the pre-post NDSE and SEES ratings for three exercise episodes, resulting in 42 ratings of response to exercise.

Demographic, metabolic, and treatment characteristics are presented in Table 1. 56.5% of the participants were diagnosed with schizoaffective disorder and 43.5% with schizophrenia. 56.5% of the participants were taking one antipsychotic medication at the time of the study, and 43.5% were prescribed more than one. Thirteen percent of the study participants used tobacco products during the study, 13% carried a diagnosis of diabetes, 22% had hyperlipidemia, and 22% had hypertension. Mean BMI was 28.9 kg/m^2^ (overweight) and most recent mean serum glucose was 102 mg/dL (borderline high). However, mean blood pressure, pulse and lipid values were all in normal range.

**Table 1:** Study participant characteristics.

**Variable**	***N* = 23**	**%**
Completion of scales	14	60.8
Male	16	69.5
**Diagnosis**		
Schizophrenia	10	43.5
Schizoaffective	13	56.5
Schizophreniform	0	0
**Antipsychotic Use**		
Participants on 1	13	56.5
Participants on >1	10	43.5
**Co-morbidities**		
Diabetes	3	13
Hypertension	5	21.7
Hyperlipidemia	5	21.7
Tobacco use	3	13
**Health parameters**	**Mean**	
BMI	28.9 kg/m^2^	
Total cholesterol	176.3 mg/dL	
HDL	57 mg/dL	
LDL	91.2 mg/dL	
Triglycerides	142.1 mg/dL	
Blood glucose	102 mg/dL	
Systolic BP	122 mmHg	
Diastolic BP	80 mmHg	
Heart rate	87 BPM	

In terms of medication treatment, 13 patients (56.5%) were on one antipsychotic medication while the other 10 (43.5%) were taking two antipsychotic medications during the study. The most common antipsychotic medications were clozapine (12 participants taking), olanzapine (5 participants taking), and aripiprazole (5 participants taking). Other participants were taking risperidone, quetiapine, paliperidone, lurasidone, and haloperidol. Finally, 2 participants were taking long acting injectable versions of antipsychotic medications: one on olanzapine and the other on paliperidone. Information on dosage was not collected. Mean pre-workout NDSE ratings ranged between 1.6 and 2.7 on psychosis and mood symptoms; 5.4–6.7 on motivation, social interest, cognition and energy items, and 3.7 on global QOL (Table 2).

**Table 2:** NDSE mean pre-workout baseline scores.

**NDSE item**	**Mean pre-workout score (cm)**	**Standard deviation**
Global QOL	3.7	2.2
Anxiety	2.2	2
Depression	2.7	2.6
Energy	5.4	2.1
Hallucinations	1.6	2.5
Delusions	1.9	2.6
Motivation	5.9	2.5
Clarity of thought	6.7	2.4
Concentration	6.4	2.5
Social interest	5.7	2.8

Participants reported an average of 4.2 exercise sessions a week with 43.5% working out for more than 60 min. Most participants (63.5%) reported working out alone. Running, weight lifting, cycling and swimming were the most popular types of exercise, with smaller proportions engaging in more diverse forms of exercise.

Participants overwhelmingly reported (91%) that the reason that they exercise is for “myself” rather than for others. Most participants (61%) had learned to exercise early in life but noted at least one period of significant disruption, usually due to an exacerbation of their mental illness. The encouragement of a doctor was helpful in re-starting exercise for 35.5% of participants. Almost seventy percent (69.6%) of participants ranked the number one reason for exercising as “self-image.” The same percentage of participants also reported psychological and physical health as very important reasons to exercise when allowed to choose more than one reason for why he or she exercised (Table 3).

**Table 3:** Semi-structured interview participant responses.

**Topic**	**Responses**	**Participants (%)**
Who do you exercise for?	Myself	91
	Others	9.1
Why do you exercise?	Self-image^a^	69.6
	Psychological health	69.6
	Physical health	69.6
	Socialization	13
	Energy	13
What improves with exercise?	Depression	56.5
	Cognitive slowing	56.5
	Anxiety	43.5
	Amotivation	26.1
	Mindfulness	17.4
	Disorganization	11.5
	Hallucinations	8.7
	Paranoia	8.7
If you do not feel like exercising, what helps?	Reminding self of benefits	30.4
	Accountability partner	26
	Reminding self of goals	17.4
	Watch TV/YouTube	13
	Nothing	13
Exercise pattern throughout life	Started young with substantial interruptions^b^	61
	Regular since childhood	34.8
	Started as adult	4.3
If there was a substantial interruption, what motivated you to re-start?	Encouraged by doctor	35.3
	Did not feel as good	23.5
	Encouraged by family/friend	23.5
	Health reasons	11.8
Current types of exercise	Running/walking	91.3
	Weight Lifting	73.9
	Cycling	52.2
	Swimming	47.8
	Elliptical/Stair-climber	34.7
	Aerobics class, Pilates, dance	21.7
	Skiing/Snowboarding	21.7
	Organized sports	13
	Hiking	13
Duration of exercise (minutes)	30–44	30.4
	45–60	17.4
	More than 60	43.5
Who do you exercise with?	No one (workout alone)	63.5
	With one or more people	36.4

a *Participants ranked this as the most important reason when asked to rank choices if multiple answers were given*.

b *The most common reason for an interruption was acute exacerbation of mental illness*.

On the SEES, study participants rated 0.6–0.2 point mean increases in feeling “Great,” “Exhausted,” “Positive,” “Terrific,” and “Strong” in response to exercise sessions (Figure 2). In addition, they rated 0.4–0.2 point mean reductions in feeling “Awful,” “Crummy,” and “Discouraged” (22).

**Figure 1:**
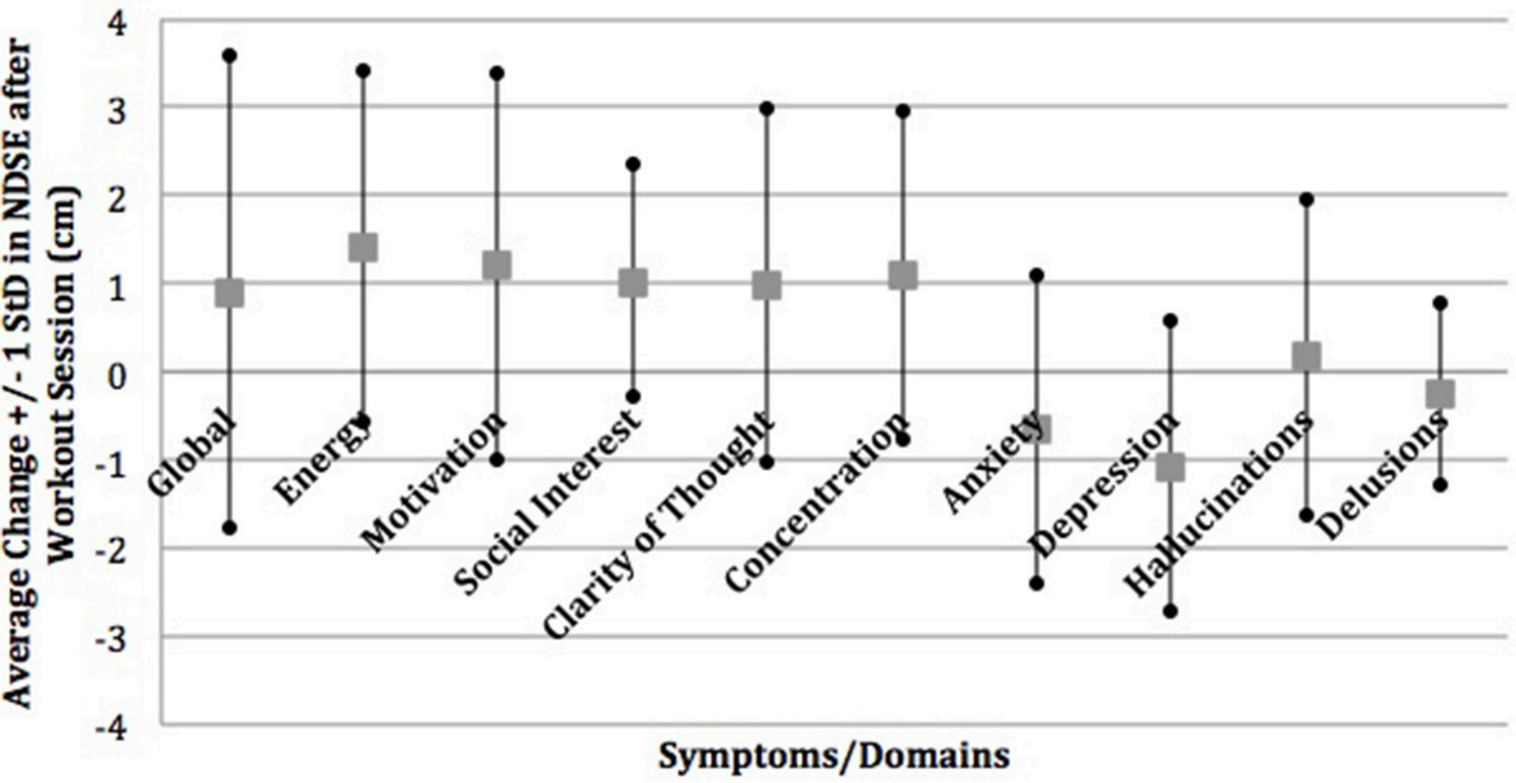
Mean change in NDSE visual analog scales. Reprinted from Dahle and Noordsy (22) with permission from Elsevier.

A majority of participants (56.5%) identified depression and cognitive slowing to be improved by exercise and 43.5% reported that exercise improved their anxiety in the semi-structured interview. This is in contrast to 8.7% of participants who identified exercise as improving psychotic symptoms such as hallucinations or paranoia. This was consistent with the prospective NDSE ratings that showed 7–11% average decreases in anxiety and depression and 10–11% average increases in clarity of thought and concentration with almost no change on average in hallucinations and delusions in response to individual exercise sessions (Figure 1) (22). NDSE ratings also showed participants rated 9–15% average increases in global QOL, energy, motivation, and social interest with each exercise session (22).

**Figure 2:**
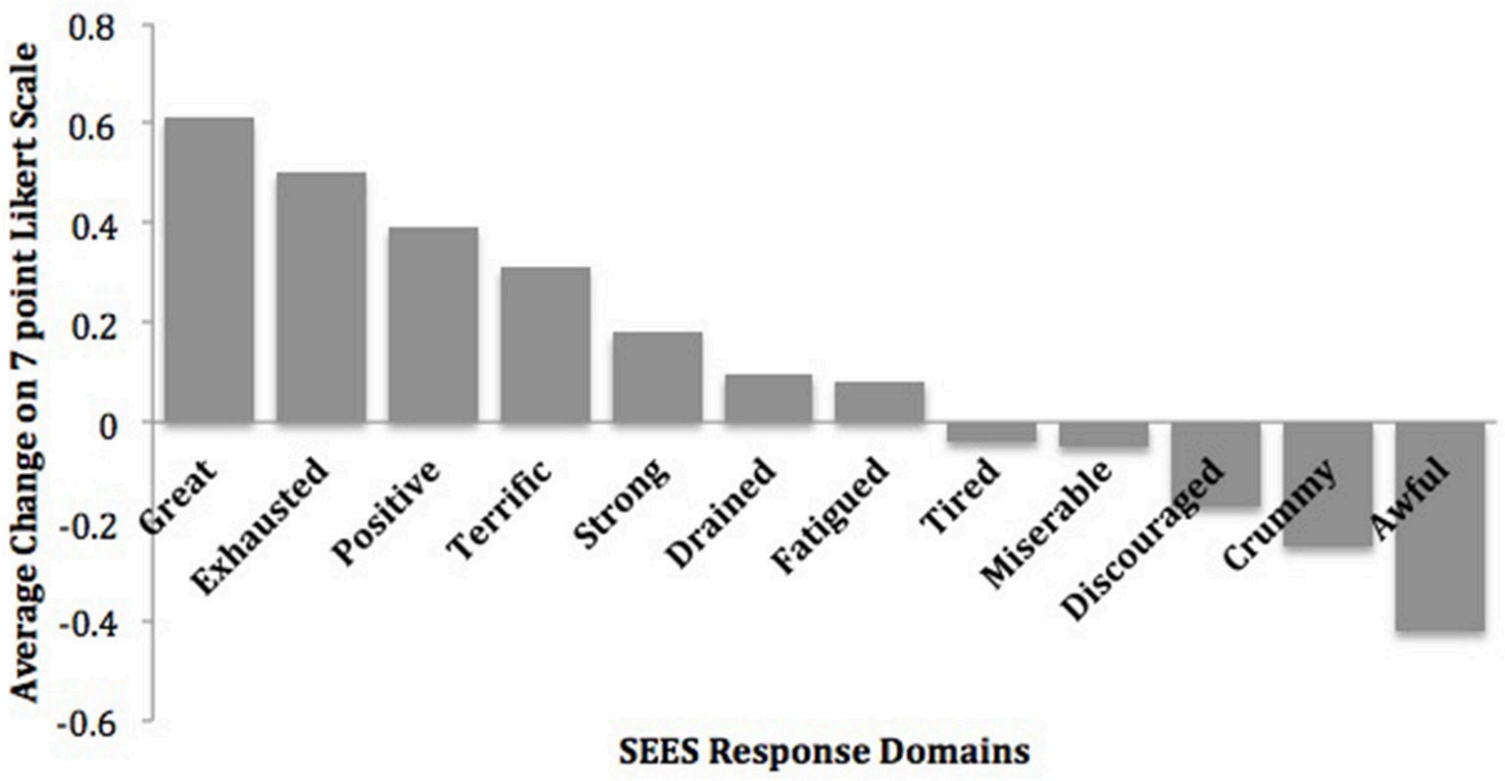
Mean change in subjective exercise experiences scale. Reprinted from Dahle and Noordsy (22) with permission from Elsevier.

## Discussion

Evidence pertaining to the effects of exercise in individuals with schizophrenia-spectrum disorders is growing. Typical designs, however, evaluate the effect of a time-limited exercise intervention on individuals who are sedentary at baseline. This study examined factors motivating and sustaining exercise in a unique population of individuals with schizophrenia-spectrum disorders who were already independently and regularly exercising. This study also examined whether self-reported retrospective mental health effects of exercise were consistent with prospective ratings at the time of actual exercise sessions.

A recent systematic review found that people with schizophrenia engage in “significantly less” exercise than controls without schizophrenia, with about half of people with schizophrenia meeting a recommended 150 min of moderate physical activity per week (23). Our selected sample engaged in at least 90 min of exercise per week and reported an average of 4.2 sessions of running, weight lifting, cycling, swimming and other forms of exercise per week, with a majority reporting more than 45 min and nearly half reporting more than 60 min of exercise per session. Taken together, these findings indicate that while people with schizophrenia can stereotypically be considered inactive, some individuals engage in types and durations of exercise that would be considered exceptional in the general population.

Our study showed that individuals with schizophrenia-spectrum disorders were most commonly motivated to exercise for “self-image” or, as one participant stated, “to look good in jeans.” This is noteworthy, as overemphasis by the medical community on the physical and mental health benefits when attempting to motivate patients to add a routine of physical exercise may be misguided. This motivation is consistent with past research on motivation for exercise in other populations. As noted by Brudzynski and Ebben in a study of body image and exercise in a midwestern university population, “appearance, was identified as the most common contributor to individual exercise behaviors” (24). While the patients in our study did identify physical and mental health reasons as motivators for exercising, these were neither the primary nor the most important reasons. Furthermore, the finding that most of this unique population developed regular exercise habits during youth suggests the importance of culture and community in shaping healthy habits that can be sustained despite a diagnosis of major mental illness. It also shows the important role that physicians can play in encouraging patients to resume regular exercise after a disruption due to an acute exacerbation of their illness.

Our finding that people with schizophrenia report improved global well-being, depression, anxiety, energy, motivation, and cognition is consistent with prior research on mental health effects of exercise across multiple populations (25). Effects on subjective energy are mixed and may represent differences in impact on physical and mental energy. The finding of improved mood, negative, and cognitive symptoms with limited impact on positive symptoms is consistent specifically with prior research among people with schizophrenia (26). Although the effects were subjective and modest, these findings were consistent across both semi-structured interviews and structured prospective ratings.

Our sample reported low mean levels of psychosis and mood symptoms, moderate levels of motivation, social interest, cognition and energy and slightly favorable QOL immediately prior to their naturalistic exercise sessions. While population norms have not been established for the NDSE and there was substantial variability, these ratings suggest that our study participants were not highly symptomatic prior to exercise. This could mean that independent exercise represents a marker of high functioning, or be due to accrued benefits of their exercise activity. It is also possible that participants chose to exercise at times when they were less symptomatic or experienced anticipatory improvement in symptoms and well-being prior to exercising.

We found modest changes on the SEES and NDSE ratings of mental health parameters from immediately before to immediately after individual sessions of exercise. It is logical that single session effects would be smaller than cumulative effects over months of exercise. Given that respondents engaged in 30–60 + min of exercise per session, these effects are occurring quite rapidly. Participants were selected for sustained exercise, so they have likely accrued gains in their mental health prior to participation. These measures are targeting the time-limited effects of exercise that fade prior to the next exercise session. Therefore, the semi-structured interview provides evidence on the global effects of sustained exercise over individuals' moderate to long-term awareness, while the SEES and NDSE ratings hone in on the immediate reinforcing effects of individual exercise sessions. This suggests that benefits of exercise in non-psychotic symptom domains are subject to decay in the 1–2 day period between exercise sessions.

A meaningful subset of participants did report improvement in symptoms of psychosis in response to exercise in the interview, which was not reflected in the mean NDSE ratings. This suggests that the impact of exercise on the subjective experience of psychosis may be more gradual and sustained. It is also possible that the subset that reported improvement in psychosis were less likely to return completed NDSE ratings, or that low baseline levels of psychotic symptoms created floor effects.

The biological effects of vigorous sustained physical activity or exercise on the human body are numerous and include cardiovascular, pulmonary, metabolic, musculoskeletal, and immunologic regulation, as well as, changes in brain functioning and anatomy (27). In people with psychiatric disorders, no single mechanism has been found to account for the diverse range of health effects of exercise. Several have been suggested: (1). biochemical changes such as increased levels of neurotransmitters (e.g., endorphins or serotonin) or altered stress reactivity (hypothalamus-pituitary-adrenal axis), (2). physiological changes such as improved cardiovascular function or thermogenesis, and (3). psychological changes such as social support, sense of autonomy, experience of mastery, enhanced body image, self-efficacy, and improved coping skills (e.g., use of distraction) (25, 28, 29). One mechanism that has been proposed for people with schizophrenia is exercise-induced neurogenesis and synaptic proliferation. A study by Pajonk et al. found exercise-induced increases in hippocampal volume and decreases in positive and negative symptoms within 3 months, and another by Scheewe et al. found increases in gray matter volume and cortical thickness by 6 months (16, 30). While there is some evidence to suggest that neurogenic effects may develop relatively quickly in response to exercise, meaningful effects on mental and physical health will likely require sustained lifestyle changes (13).

Our study was limited by several factors. Our study relied on self-report and the use of less structured probes for the interview portion of the assessment. Interviewers may have elicited responses that participants might not have offered spontaneously. Validated measures of negative symptoms rely heavily on rater observation and it is not clear that people can accurately self-report this construct. Our study population was small, and recruited from a rural sample of patients. Although this convenience sample may not be representative of people with schizophrenia in other settings, all patients from the authors' usual care clinics who met criteria and consented to participate within the recruitment window were included. Another limitation is that the NDSE is not yet validated (a validation study is underway). Our participants, however, found the NDSE easy to use and demonstrated an expected degree of responsiveness to individual exercise sessions. Their responses on the NDSE closely paralleled their responses on the well-established SEES.

## Conclusions

To our knowledge, this is the first study to examine a cohort of active and community dwelling individuals diagnosed with schizophrenia in order to describe their exercise patterns and subjective experiences. The granular detail on effects of individual exercise sessions provides a unique view into the experience of exercise for people living with schizophrenia. Our findings establish that some individuals with schizophrenia engage in regular physical exercise that stem from earlier life activities. These individuals identify primary motivations similar to those of people who do not have schizophrenia. Duration and frequency of exercise rival those of community amateur athletes, and mean metabolic parameters are largely healthy despite BMI in overweight range. Individual sessions of exercise are rapidly reinforced by improvements in several areas including well-being, energy, and non-psychotic symptoms.

## Ethics statement

This study was carried out in accordance with the recommendations of the institutional review board of Dartmouth-Hitchcock Medical Center with written informed consent from all subjects. All subjects gave written informed consent in accordance with the Declaration of Helsinki. The protocol was approved by the institutional review board of Dartmouth-Hitchcock Medical Center.

## Author contributions

DD and DN contributed to study design and implementation. All authors contributed to manuscript preparation and approved the final draft.

### Conflict of interest statement

The authors declare that the research was conducted in the absence of any commercial or financial relationships that could be construed as a potential conflict of interest.
